# Impact of acetate- or citrate-acidified bicarbonate dialysate on ex vivo aorta wall calcification

**DOI:** 10.1038/s41598-019-47934-7

**Published:** 2019-08-06

**Authors:** Ricardo Villa-Bellosta, Eduardo Hernández-Martínez, Eva Mérida-Herrero, Emilio González-Parra

**Affiliations:** 1 Fundación Instituto de Investigación Sanitaria, University Hospital Fundación Jiménez Díaz (FIIS-FJD), Universidad Autónoma de Madrid, Renal Division, Avenida Reyes Católicos 2, 29040 Madrid, Spain; 2 Renal Division, University Hospital 12 de Octubre, Avenida de Córdoba, s/n, 28041 Madrid, Spain

**Keywords:** Ageing, Calcification

## Abstract

Vascular calcification is highly prevalent in patients with chronic hemodialysis. Increased acetatemia during hemodialysis sessions using acetate-acidified bicarbonate has also been associated with several abnormalities, By contrast, these abnormalities were not induced by citrate-acidified bicarbonate dialysis. Moreover, citrate is biocompatible alternative to acetate in dialysis fluid. However, the effects of citrate on vascular calcification during hemodialysis had not been studied in detail. This study analyzed herein the effects of acetate- or citrate-acidified bicarbonate dialysis on vascular calcification. Citrate has been shown to inhibit calcification in urine in hemodialysis patients. Therefore, our hypothesis is that citrate-acidified bicarbonate dialysis could reduce vascular calcification. Blood samples before and after hemodialysis from patients on acetate- or citrate-acidified bicarbonate dialysis were collected in heparin-containing tubes (n = 35 and n = 25 respectively). To explore the effect of pre- and post-dialysis plasmatic bicarbonate and citrate on vascular calcification, rats aortic rings cultured *ex vivo* in Minimum Essential Medium containing 0.1% FBS and 45-calcium as radiotracer were used (n = 24). After 7 days of incubation aortic rings were dried, weighed and radioactivity was measured via liquid scintillation counting. Bicarbonate levels increase calcium accumulation in rat aortic wall in a dose-response manner (pH = 7.4). Moreover, citrate prevents calcium accumulation, with a mean inhibitor concentration (IC_50_) value of 733 µmol/L. During acetate-acidified bicarbonate dialysis, bicarbonate and citrate levels in plasma increase (22.29 ± 3.59 versus 28.63 ± 3.56 mmol/L; p < 0.001) and decrease (133.3 ± 53.6 versus 87.49 ± 32.3 µmol/L, p < 0.001), respectively. These changes in pos-hemodialysis plasma significantly (p < 0.001) alter calcium accumulation in the aortic wall (38.9% higher). Moreover, citrate-acidified bicarbonate dialysis increases post-hemodialysis citrate levels 5-fold (145 ± 79.8 versus 771.6 ± 184.3 µmol/L), reducing calcium accumulation in the aortic wall. Citrate-acidified bicarbonate dialysis reduces plasmatic calcium and pH variations during dialysis session (Δ[Ca^2+^] = −0.019 ± 0.089; ΔpH = 0.098 ± 0.043) respect to acetate-acidified bicarbonate dialysis (Δ[Ca^2+^] = 0.115 ± 0.118; ΔpH = 0.171 ± 0.078). To our knowledge, our study is the first to show that citrate protects against calcium accumulation in rat aortic walls *ex vivo*. Therefore, citrate-acidified bicarbonate dialysis may be an alternative approach to reduce calcification in hemodialysis patients without additional cost.

## Introduction

Vascular calcification, a predictor of cardiovascular disease and all-cause mortality^1^, is frequently observed in chronic hemodialysis patients, mainly because serum phosphorus and calcium levels are frequently higher in these patients^2^. Hydro-electrolyte disturbances that occur during hemodialysis increase the risk of vascular calcification due to incremental increases in calcium concentration^3^, alkaline phosphatase activity, and alkalosis^4^. To prevent magnesium carbonate and calcium carbonate precipitation in dialysis fluid containing bicarbonate, an acid, such as acetate, is usually added, but causes side effects. Increased acetatemia during hemodialysis sessions using acetate-acidified bicarbonate (currently the most used) has been associated with several abnormalities, including increases in pro-inflammatory cytokines and oxidative stress. Moreover, hydro-electrolyte disturbances that occur during hemodialysis increase the risk of vascular calcification due to incremental increases in calcium concentration and alkalosis.

Citrate-acidified bicarbonate dialysis uses citrate instead and is considered more biocompatible, safer, and better at preventing severe acid-base and calcium illnesses^5,6^ than acetate Additionally, acetate-acidified bicarbonate can be replaced by citrate-acidified bicarbonate at no extra cost.

However, the effects of using citrate in the dialysate on the development of vascular calcification are unclear and have been poorly studied. This study compared the effects of acetate- and citrate-acidified bicarbonate dialysis on vascular calcification. Finally, because citrate inhibits calcification in hemodialysis patient urine, we determined whether citrate-acidified bicarbonate dialysis reduces vascular calcification in these patients.

## Methods

### Hemodialysis conditions and sampling

Each patient underwent a conventional, purely diffusive 4 hour (mid-week) hemodialysis session without hemodiafiltration, using a high flux helixone dialyzer (Fresenius, CUF, 59 mL/h/mmHg; surface, 1,8 m^2^) and Dialysis Machine Fresenius 5008 in acetate-acidified bicarbonate dialysis, according to previous studies^3,4,7^. In case of citrate-acidified bicarbonate dialysis, high flux helixone dialyzer (Polyflux 21H, Gambro, CUF 78 ml/min/ mmHg, surface 2.1 m^2^) and Dialysis Machine Artis Baxter were used. The acetate-acidified bicarbonate dialysate was composed of 3–4 mmol/L acetate, 1.5 mmol/L calcium, 35 mmol/l bicarbonate, 1.5 mmol/L potassium, 0.5 mmol/L magnesium and 140 mmol/L sodium. The citrate-acidified bicarbonate dialysate (SelectCitrate Cx250G, Gambro) contained 1 mmol/L citrate without acetate, 1.65 mmol/L calcium, 35 mmol/l bicarbonate, 2 mmol/L potassium, 0.5 mmol/l magnesium and 140 mmol/L sodium).

Blood samples before and after hemodialysis were collected in heparin-containing tubes and centrifuged at 5000 rpm for 5 min at 4 °C, according to previous studies^3,4,7^. Plasma samples were frozen in liquid nitrogen and stored at −80 °C until further use, as previously studies^3,4^. This study was conducted according to the Declaration of Helsinki and was approved by the Ethics Committees of Research of University Hospital Fundación Jiménez Díaz (Madrid, Spain) and University Hospital 12 de Octubre (Madrid, Spain). Participants were identified by a number and no other identifying material. Subject were included if they were adults on stable chronic hemodialysis with a life expectancy over 6 months according to clinical criteria and provided informed consent. Patients with positive serology for HIV, HB surface Ag, or HCV or other known active infection were excluded, according to previous studies^3,4,7^.

Bicarbonate, ionic calcium and pH variables were measured using an ADVIA CENTAUR 2400 autoanalyzer according to the manufacturer’s protocols. Citrate was measured using Citrate assay kit (MAK057-1KT, Sigma-Aldrich) according to the manufacturer’s protocols. Samples were conducted in triplicate.

### Aorta isolation and calcification assay

Male Sprague-Dawley Rats were obtained from Charles River Laboratories (France). The protocol was approved by ethics committees of both Fundación Instituto de Investigación Sanitaria, Fundación Jiménez Díaz and Madrid Community (PROEX 427/15) and conformed to directive 2010/63EU and recommendation 2007/526/EC on the protection of animals used for experimental and other scientific purposes, enforced in Spanish law under RD1201/2005, according to previously studies^3,7,8^^,^.

Rats were euthanized via carbon dioxide inhalation and thoracic aorta tissue was perfused with saline and removed according to previously published protocols^8^. For calcification assays, aortic rings were cultured *ex vivo* (37 °C. 5% Co2) in Minimum Essential Medium (MEM) Eagle (Gibco, Paisley, United Kingdom) containing 45-calcium as a radiotracer (Perkin Elmer, Boston), 0,1% Fetal Bovine Serum, 1 mmol/L L-glutamine, 100 IU/mL, penicilin, 100 µg/mL streptomycin and 0.1% fetal bovine serum^7^; and supplemented with indicated concentration of citrate and bicarbonate. After 7 days of incubation aortic rings were dried, weighed and radioactivity was measured via liquid scintillation counting (Perkin Elmer Tri-Carb 2810TR). The half maximal inhibitor concentration (IC_50_) was calculated with GraphPad Prism 5 software by nonlinear regression using the classical 1-site competition equation, C = bottom + ([top-bottom]/[1–10^(S-logIC50)^]), according previously studies^8^. C refers to the calcification (calcium content in the aortic rings); IC_50_ refers to the concentration of citrate that is required for 50% inhibition of calcification; Top refers to the calcification in the absence of citrate and Bottom refers to maximum inhibition of the calcification. Points represent 24 rat aortic rings (three independent experiments, each using eight rings per condition).

### Statistical analyses

The Kolmogorov-Smirnov test was used to assess the normality of the data. Results are presented as means ± SD. For calcification assays, the number of experiments is indicated in the figure legends. In all cases, statistical significance was assigned at p < 0.05. GraphPad Prism 5 software was used for statistical analysis.

## Results

### Bicarbonate dose-dependently increases calcium accumulation in rat aortic rings

**Figure 1:**
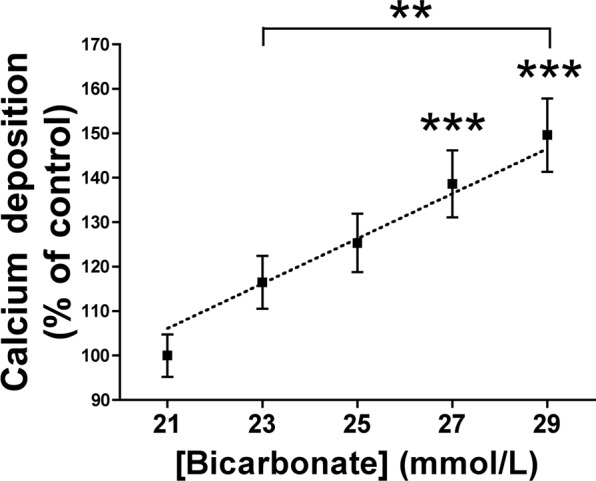
Bicarbonate increases calcium accumulation in rat aortic wall. Rat aortic rings were cultured *ex vivo* (37 °C. 5% CO_2_) in Minimum Essential Medium Eagle containing 45-calcium as a radiotracer and the indicated concentrations of bicarbonate (pH = 7.4). After 7 days of incubation, the aortic rings were dried and weighed. Radioactivity was measured by liquid scintillation counting. Results represent three independent experiments, each using eight rings per condition. **P < 0.01; ***P < 0.001 by one-way ANOVA and Tukey multiple comparison post hoc test.

Rat aortic rings incubated for 7 days in MEM containing different concentrations of bicarbonate (pH 7.4) showed dose-dependent increases in calcium accumulation (Fig. 1). Incubation with 27 and 29 mmol/L bicarbonate resulted in significantly greater (p < 0.001) calcium accumulation that incubation with control solution (21 mmol/L bicarbonate). In addition, incubation with 29 mmol/L bicarbonate resulted in significantly greater calcium accumulation (p < 0.001) than incubation with 23 mmol/L bicarbonate.

### Citrate reduces calcium accumulation in rat aortic rings

**Figure 2:**
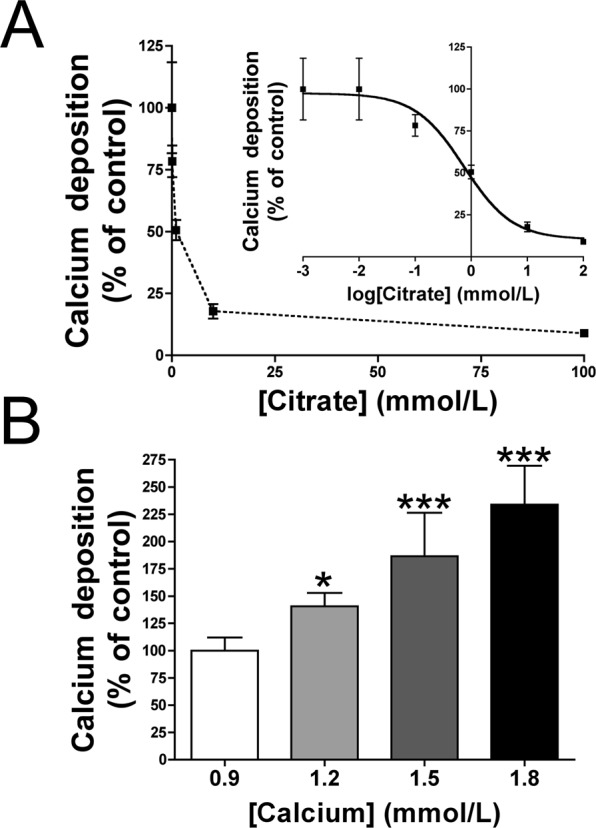
Citrate prevents calcium accumulation in rat aortic wall. Rat aortic rings were cultured *ex vivo* (37 °C. 5% CO_2_) in Minimum Essential Medium Eagle containing 45-calcium as a radiotracer and the indicated concentrations of citrate. After 7 days of incubation, the aortic rings were dried and weighed. Radioactivity was measured by liquid scintillation counting. (**A**) Effect of citrate concentration on calcium accumulation in aortic walls. (**B**) Effect of calcium concentration on calcium accumulation in the presence of a constant concentration of citrate (733 µmol/L). Results represent three independent experiments, each using eight rings per condition. *P < 0.05; ***P < 0.001 by one-way ANOVA and Tukey multiple comparison post hoc test.

Incubation for 7 days in MEM containing 1.1 mmol/L calcium and the indicated concentration of citrate (pH 7.4) prevented calcium accumulation in rat aortic rings, with an IC_50_ of 733.2 µmol/L (Fig. 2A). Moreover, at a constant citrate concentration (733 µmol/L), calcium accumulation correlated positively and dose-dependently with calcium concentration (Fig. 2B).

### Effects of acetate-acidified bicarbonate dialysis on plasma bicarbonate/citrate levels and on aortic calcium accumulation

**Figure 3:**
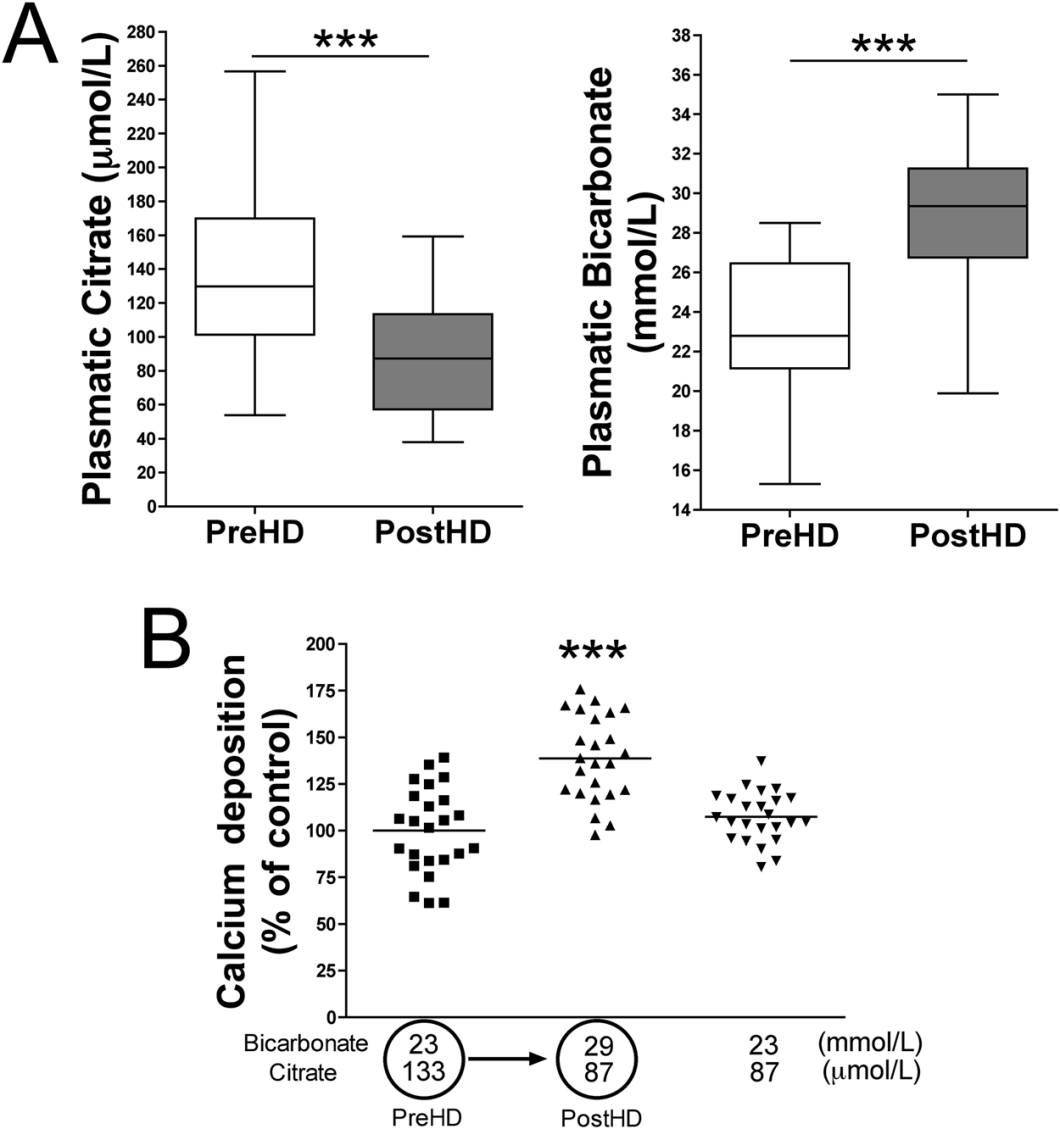
Effects of acetate-acidified bicarbonate dialysis on rat aortic wall calcification. (**A**) Concentrations of the indicated metabolites in plasma before (Pre-HD) and after (Post-HD) acetate-acidified hemodialysis. (**B**) Calcium accumulation in rat aortic rings incubated with citrate and bicarbonate concentrations representative of plasma before and after hemodialysis using acetate-acidified bicarbonate dialysate. Results represent three independent experiments, each using eight rings per condition. ***P < 0.001 by one-way ANOVA and Tukey multiple comparison post hoc test.

Plasma bicarbonate levels and pH were significantly higher after than before hemodialysis in all pairs of samples tested (n = 35; Table 1). Plasma calcium levels were also significantly higher after than before hemodialysis. Moreover, plasma citrate concentrations were significantly lower (p < 0.001) after (87.49 ± 32.3 µmol/L) than before (133.3 ± 53.6 µmol/L) hemodialysis in all pairs of samples (Table 1). Finally, incubation of rat aortic rings in MEM containing 29 mmol/L bicarbonate and 87 µmol/L citrate, the post-dialysis conditions in acetate-acidified bicarbonate dialysis, significantly increased (p < 0.001) calcium accumulation compared with incubation under pre-hemodialysis conditions (23 mmol/L bicarbonate and 133 µmol/L citrate; Fig. 3).

**Table 1:** Concentrations of the indicated metabolites in plasma before (Pre-HD) and after (Post-HD) acetate- and citrate-acidified hemodialysis.

	Acetate-acidified bicarbonate dialysate (n = 35)		p	Citrate-acidified bicarbonate dialysate (n = 25)		p
	Pre-HD	Post-HD		Pre-HD	Post-HD	
pH	7.276 ± 0.065	7.447 ± 0.055	<*0,001*	7.334 ± 0.066	7.431 ± 0.053	<*0,001*
Ionized Ca^2+^ (mmol/L)	1.09 ± 0.09	1.20 ± 0.09	<*0,001*	1.15 ± 0.09	1.13 ± 0.06	*0.317*
Bicarbonate (mmol/L)	23.29 ± 3.59	28.63 ± 3.56	<*0,001*	21.82 ± 2.34	27.05 ± 2.15	<*0,001*
Citrate (µmol/L)	133.3 ± 53.6	87.49 ± 32.3	<*0,001*	145.1 ± 79.8	771.6 ± 184.3	<*0,001*

Results are presented as mean ± S.D.

### Effects of citrate-acidified bicarbonate dialysis on plasma bicarbonate/citrate level and on aortic calcium accumulation

Plasma bicarbonate levels and pH were significantly higher after than before hemodialysis in all pairs of samples tested (n = 25; Table 1). Interesting, plasma citrate concentrations were significantly higher after (771.6 ± 184.3 µmol/L) than before (145.1 ± 79.8 µmol/L) hemodialysis in all pairs of samples (Table 1). By contrast, plasma calcium concentrations were slightly low (but not significantly different; p = 0.317) after than before hemodialysis (Table 1).

**Figure 4:**
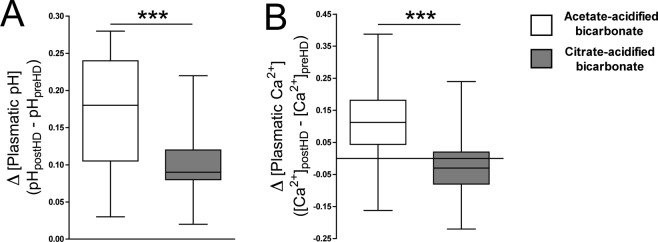
Changes in pH and ionized calcium levels using acetate- or citrate-acidified bicarbonate dialysis. Box and whiskers representing the variation both in pH **(A)** and in plasmatic ionized calcium levels (**B**) during dialysis session using acetate-acidified bicarbonate (white box) or citrate-acidified bicarbonate (gray box). ***P < 0.001 by Student *t* test. PreHD: pre-hemodialysis; PostHD: post-hemodialysis.

The increment in bicarbonate levels after dialysis session (Δ[bicarbonate] = [bicarbonate]_post-hemodialysis_ − [bicarbonate]_pre-hemodialysis_) were slightly low using citrate-acidified bicarbonate (Δ[bicarbonate] = 5.23 ± 2.86) than acetate-acidified bicarbonate dialysis (Δ[bicarbonate] = 5.62 ± 3.08), but not statistically different (p = 0.484). However, the pH variation during dialysis session (ΔpH = pH_post-hemodialysis_ − pH_pre-hemodialysis_) were significantly low (p < 0.001) using citrate-acidified bicarbonate (ΔpH = 0.098 ± 0.043) than acetate-acidified bicarbonate dialysis (ΔpH = 0.171 ± 0.078; Fig. 4). Moreover, the changes in plasmatic ionized calcium levels during dialysis session (Δ[Ca^2+^] = [Ca^2+^]_post-hemodialysis_ − [Ca^2+^]_pre-hemodialysis_) were significantly low (p < 0.001) using citrate-acidified bicarbonate (Δ[Ca^2+^] = −0.019 ± 0.089) than acetate-acidified bicarbonate dialysis (Δ[Ca^2+^] = 0.115 ± 0.118; Fig. 4).

**Figure 5:**
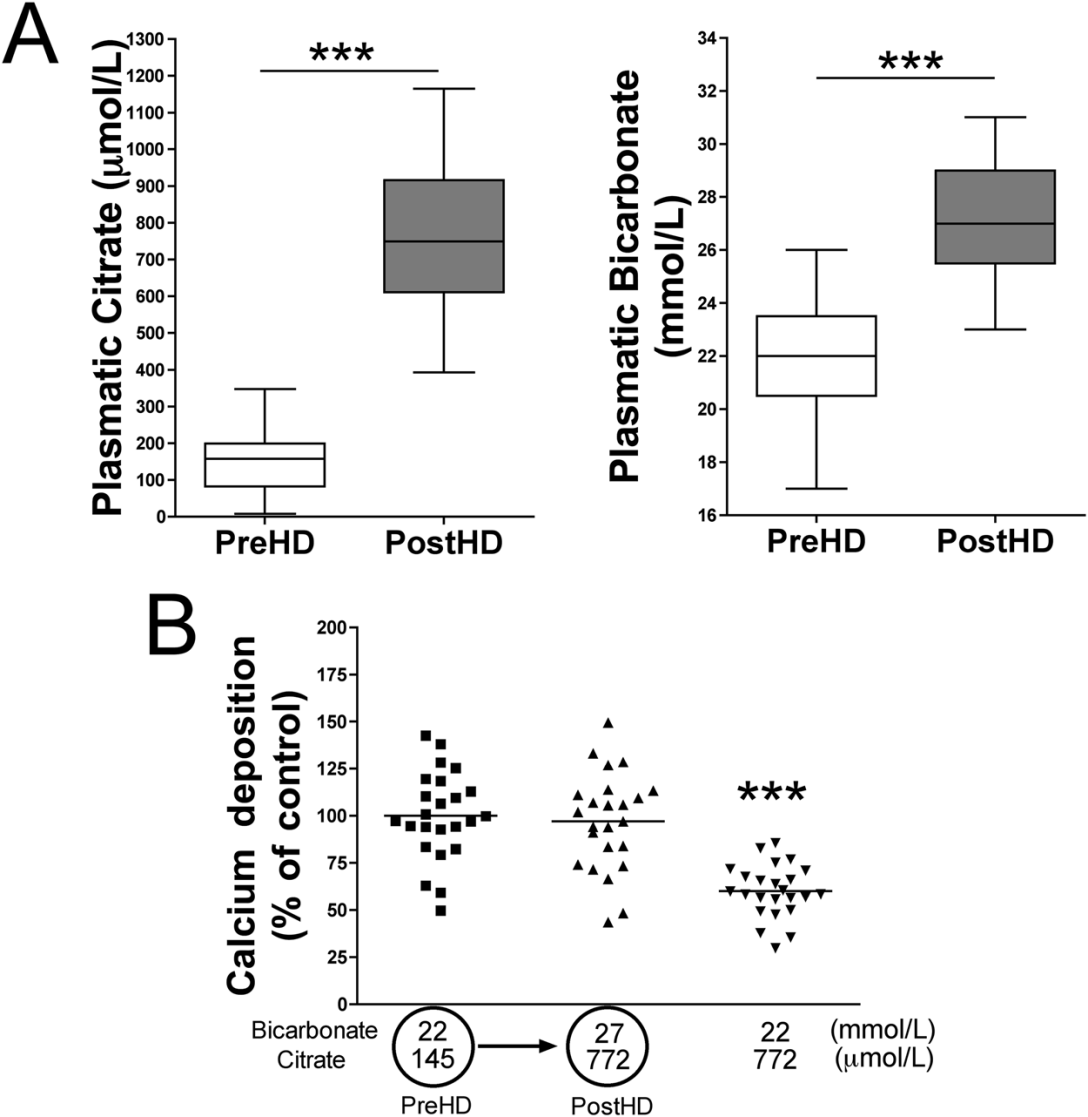
Effects of citrate-acidified bicarbonate dialysis on rat aortic wall calcification. (**A)** Concentrations of the indicated metabolites in plasma before (Pre-HD) and after (Post-HD) citrate-acidified hemodialysis. (**B**) Calcium accumulation in rat aortic rings incubated with citrate and bicarbonate concentrations representative of plasma before and after hemodialysis using citrate-acidified bicarbonate dialysate. Results represent three independent experiments, each using eight rings per condition. ***P < 0.001 by one-way ANOVA and Tukey multiple comparison post hoc test.

Finally, incubation of rat aortic rings in MEM under post-dialysis conditions of citrate-acidified bicarbonate dialysis (27 mmol/L bicarbonate and 772 µmol/L citrate) reduced calcium accumulation when compared with pre-hemodialysis conditions (22 mmol/L bicarbonate and 145 µmol/L citrate; Fig. 5).

## Discussion

Increased acetatemia during hemodialysis sessions using acetate-acidified bicarbonate has been associated with several abnormalities, including increases in oxidative stress, pro-inflammatory cytokines and nitric oxide synthesis. By contrast, these abnormalities were not induced by citrate-acidified bicarbonate dialysis^9^. Other studies also show the advantages and safety of citrate-acidified bicarbonate dialysis. For example, citrate is biocompatible alternative to acetate in dialysis fluid^10^, and bicarbonate dialysate acidified with citrate increases solute removal^11^. Moreover, citrate has been used as a phosphate binder during hemodialysis, for treatment of hyperphosphatemia and as an anticoagulant^12–14^. Citrate has been also shown to inhibit calcification in urine in patients with chronic kidney disease^15^, and potassium citrate has been recommended to treat nephrocalcinosis^16^. However, the effects of citrate on vascular calcification during hemodialysis had not been studied in detail.

To our knowledge, our study is the first to show that citrate protects against calcium accumulation in rat aortic walls incubated *ex vivo*. For example, citrate had an IC_50_ of 733 µmol/L in preventing calcification induced by 1.1 mmol/L calcium. This protective effect was inversely related to calcium concentration. Interestingly, citrate concentrations in plasma were lower following acetate-acidified bicarbonate dialysis but were increased 5-fold following citrate-acidified bicarbonate dialysis.

We also found that bicarbonate dose-dependently increased calcium accumulation in the aortic wall. Because bicarbonate is included in both citrate- and acetate-acidified bicarbonate dialysate the plasmatic concentration of bicarbonate increases after dialysis session similarly in both groups, although citrate can be degraded to bicarbonate^17^. Therefore, following acetate-acidified bicarbonate dialysis the increase in bicarbonate and the reduction in citrate levels increased calcium accumulation in the aortic wall. By contrast, citrate-acidified bicarbonate dialysis increased the concentrations of both bicarbonate and citrate, with no change in calcium accumulation in the aortic wall. Therefore, citrate-acidified bicarbonate dialysis may be an advantageous alternative to acetate-acidified bicarbonate dialysis, preventing vascular calcification^18^.

In the other hand, citrate-acidified bicarbonate dialysis significantly reduces the post-dialysis increments both in pH and in plasmatic ionized calcium levels (Table 1 and Fig. 4), two know vascular calcification enhancers; and thus, this dialysis type also improves the risk of vascular calcification induced by increments both in plasma calcium and pH after dialysis session^3,4^.

The study has a limitation that should be acknowledged. Rat aortic rings where used to assess the effect of bicarbonate and citrate on the aortic wall calcification *ex vivo*. Therefore, translational studies in human subjects are required, including the assessment of vascular calcification between to equivalent group of patients receiving citrate or acetate based dialysis. Our study may provide sufficient molecular proof to initiate long-term studies comparing vascular calcification following acetate- and citrate-acidified bicarbonate dialysis in a large number of patients undergoing hemodialysis.
